# Our Next Volume

**Published:** 1855-05

**Authors:** 


*Our next Volume.*—The valedictory remarks of our colleague, and the circular mailed with this number, will inform our readers of the change which has taken place in the management of this Journal—a change of men, not principles. We hope that in losing our colleague we are not to lose his old friends. Those old subscribers so long accustomed to his writings will still find his name at the head of an occasional article.

Time will prove how many of the anticipations with which we enter upon our task will be fulfilled. We assume the ownership of the Journal with some acquaintance with the wants of its readers, and with the determination to make it a paying affair to them and to ourselves. We are, at any rate, safe from loss, and we mean, if time and labor on our part can ensure success, to keep the Journal, what it ever has been, a good one; and to find our reward in an extended subscription list. H.

